# The Natural Fallacy in a Post‐Truth era: A perspective on the natural sciences’ permeability to values

**DOI:** 10.15252/embr.201949859

**Published:** 2020-01-10

**Authors:** Mariana RP Alves

**Affiliations:** ^1^ European Molecular Biology Laboratory Heidelberg Germany

**Keywords:** S&S: Ethics, S&S: History & Philosophy of Science

## Abstract

The natural sciences are not immune to societal values and beliefs. Understanding the interference of these factors is key to protect science from becoming a servant to opportunistic interests.

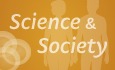

Understanding the permeability of science to values—beliefs, prejudices, preferences or convictions—is of great importance for science itself. On the one hand, the interference of values challenges the objectivity of science; on the other hand, science is being increasingly used and instrumentalized to legitimize and enact public policies based on values. Yet, the role of values in the natural sciences is currently underrated, as most of research on this topic has been done by and for the social sciences and humanities. The debate around facts versus values is not new in the history of science and philosophy. In the 18^th^ century, philosopher David Hume argued in *A Treatise of Human Nature* that it is a crucial discussion to inform scientific practice. It is nonetheless essential to understand value interference in the natural sciences to ensure that research will not become the servant of opportunistic interests, legitimizing actions that can have a negative impact on society. Moreover, being aware of this interference is a key to best practices in science.

According to philosopher Hans Reichenbach, scientific contexts can be divided into two categories: the context of discovery and the context of justification. The first is associated with the formulation of theories, which can be influenced by historical, social or psychological factors. The context of justification is associated with determining the truth or falsity of a theory. Values can interfere with scientific practice—or with its so‐called “neutrality” or “objectivity”—in both contexts as what philosopher Sampaio da Silva described as epidermal and hypodermic contaminations, respectively 1. da Silva and other philosophers have argued that the real threat to science's neutrality is the interfere of values with the context of justification, that is the determination of the truth or falsity of a theory, whereas the choice of hypothesis, methods or topics in the context of discovery does not compromise the objectivity of science 1. However, the context of discovery also deserves special attention; Max Weber argued that the influence of dominant societal values is pivotal in deciding what becomes the subject of investigation and what does not (Revised in ref. 2).

It is nonetheless essential to understand value interference in the natural sciences to ensure that research will not become the servant of opportunistic interests…

Despite frequent and intense clashes between social scientists’ theories and perspectives—which are symptomatic of the role of values in science—social scientists, for a long time, resisted to admit the lack of neutrality of their discipline. Today, the discussion on values’ interference is still ongoing in the social sciences 2, whereas the natural sciences have been avoiding this topic under the shield of the “exact sciences”.

## Lessons from the social sciences

One example from the social sciences of how values interfere with science is the subdetermination of a theory by data. This is the idea that, from the same data, one can build different theories that are logically incompatible with each other while still empirically equivalent. This is a relevant argument for the natural sciences too, particularly in an era of “data‐driven” research. It can happen when data are being generated without an underlying hypothesis, and scientists try to come up with justifications for generating these data in the first place—quoting Sydney Brenner, “low input, high throughput, no output science”.

A well‐known example from the social sciences is intentional and functional explanations for the behaviour of German citizens who participated in the assassination of Polish Jews during the Holocaust. The intentional explainers argue that human behaviour is characterized by beliefs and that Germans’ behaviour originated from anti‐Semitic beliefs ingrained in their culture. The functional explainers argue that human behaviour is characterized by its circumstances and that the atrocities committed during the Holocaust had originated from general societal problems such as obedience to authority and peer pressure. Supporting either of these theories and their methodologies is greatly influenced by values and context. This example also shows how theories with completely different implications can be built from the same data and study, depending on which methodological angle is being used.

Another example from the social sciences is the issue of studying a societal problem, such as anti‐vaccine beliefs, in a community that does not identify this behaviour as a problem. In this case, scientists inevitably use other, social values to justify characterizing these social phenomena as a problem that requires study.

Several arguments and examples show that natural scientists are not immune from social values either and are not working in an environment of absolute objectivity and neutrality. This is a very important topic to discuss since innovation and discovery can be severely stunted by dogmatic attitudes in science. And, contrary to the belief of many scientists, this is a discussion that should not to be left to philosophers.

## Arguments in the context of the natural sciences

“As is well known, in medical practice, given the potentially serious consequences of a false negative, doctors in their diagnoses give priority to the hypotheses of serious illness, because in this case, the consequences of the error are not serious. On the contrary, the judicial system prefers false negatives (the acquittal of a guilty person) to false positives (the condemnation of an innocent)” 3. But it is not just the medical or judicial system that make such value‐based decisions; natural scientists make similar choices between favouring false positives or false negatives. One example is the work of Heather Douglas on inductive risk, which assesses the consequences of accepting or rejecting a hypothesis that is associated with an error risk 4. Douglas gives examples of how not only the decision to accept or reject a hypothesis is permeable to values, but also the choice of methodologies, the collection of data, and their analysis and interpretation. This problem has been discussed extensively in the context of GMO safety regulation, and the adjacent debate about the weight of expert opinion versus empirical evidence.

But it is not just the medical or judicial system that make such value‐based decisions; natural scientists make similar choices between favouring false positives or false negatives.

Here, it is important to distinguish between internal values and external constraints that influence research, methodologies and interpretation. The number of animals used in an experiment, tests being done on human volunteers, the use of radioactive substances or technologies that can be abused for nefarious purposes are all regulated or even prevented by legal and regulatory frameworks. Drug safety and efficiency tests and their interpretation are subject to similar externally imposed laws and regulations. In contrast, the societal context influences internal values that in turn impact on scientific practice, such as the choice and formulation of hypotheses—including the choice to pursue a pet hypothesis—methods or the analysis of data. Here again, the permeability of the natural sciences to values is prevalent in statistical choices: what is deemed statistically significant differs from study to study. Consequently, we can observe a certain relativism regarding statistics: either legitimization of results that are not statistically relevant, by shielding them with inappropriate statistical criteria; or disregard of relevant findings by a general distrust on statistical choices. This relativism is associated with a decreased confidence in scientific evidence 5.

## The natural sciences and the fact‐value debate

The increasing legitimacy that society gives to science promotes a dogmatic view of scientific knowledge and aggravates the consequences of a lack of reflection about the role of values in natural sciences. An example of this dogmatic approach in popular science is the famous quote by the American astrophysicist and science communicator Neil de Grasse Tyson that “science is true whether or not you believe in it”. Natural science curricula stress the objectivity and neutrality of science and the scientific method and too often ignore topics such as the history of science or central epistemological issues that would encourage critical inflection on the influence of values and beliefs. In parallel, there is a rising appearance of scientific fraud, which results from a crisis of shared moral societal values. In addition, the presence of scientists in ethics committees at the expense of philosophers may also lead to a rather one‐sided view of what is considered to be right.

There is even the argument “of the manipulatable objectivity” 1 that denies the interference of values. It claims that scientists and science are being “manipulated” by reality as scientists cannot ignore observations and results that show a hypothesis to be false. Thus, the resulting and surviving theories are always as objective as possible and thereby immune to the interference of social or personal values. Nonetheless, even if so, it does not give scientists immunity against value contamination, nor should it be an argument against critical thinking about scientific practice and values. “The belief in the value of scientific truth is a product of certain civilizations and not a fact of nature” 6.

The increasing legitimacy that society gives to science promotes a dogmatic view of scientific knowledge…

How then can we resolve the problem that the interference of values in the natural sciences is too often ignored? How can natural scientists understand that there is no separation of their scientific activity from their human context? How can we have this debate without compromising trust in scientific evidence in times of post‐truths, post‐facts, post‐science and pseudoscience? How can science maintain its independence from social ideologies while being an activity within a social context?

## A call for action

There must be more emphasis on epistemology in universities and more debate on this topic in research centres, by scientists and by philosophers of science. PhD students should be trained as doctors of philosophy and not solely as proficient laboratory technicians. The insight that values interfere with scientific activity may, at first, cause pessimism and discrediting science when it turns out that “curiosity” which drives scientific inquiry is not neutral and objective. Nevertheless, avoiding the issue cannot be the answer. Importantly, the interference of values in science has many positive aspects that should not be ignored. It is due to values that science is a plural, diverse and inclusive activity; it is due to values that science is reflective, and it is due to values that science addresses societal developments and challenges. Since we cannot eradicate values from science, we shall not only acknowledge them but embrace the positive influences of value interference on scientific endeavour. And embracing these positive influences includes investing and advocating for more diversity and inclusion in our institutes too. “The worst thing about the fact/value dichotomy is that in practice it functions as a discussion‐ stopper, and not just a discussion‐stopper, but a thought stopper”, as the American philosopher and mathematician Hilary Putnam put it 7.

Since we cannot eradicate values from science, we shall not only acknowledge them but embrace the positive influences of value interference on scientific endeavour.

Obviously, there needs to be more critical discussion in the scientific community. The freedom to doubt is the heart of science and doubting ourselves should be an important part of it. The expansion of research and the consequent explosion in the number of scientific papers has had the usual side effect of mass production: the loss of quality control. The flaws in the system have been exposed numerous times, more recently by the Grievance Studies Hoax, when completely fake papers were accepted by peers and journals 8. The role of peer review is also currently under critical discussion owing to the dependency of scientists on journal publications and the rise and popularity of pre‐print servers. As science gets more competitive, the time to conduct thorough peer review may inevitably become less and lead to slouch or neglect that, although not ill‐intended, may have serious consequences for the quality of research.

Nevertheless, peers who can instigate critical thinking and review of scientific work are not only reviewers of publications but also colleagues, mentors and collaborators. Teams or research groups as a whole should indulge in critical review and comments to improve each other's work and ultimately the work of the collective. From laboratory meetings to departmental seminars, to one‐on‐one mentoring—any form of scientific discussion is a helpful control mechanism for our permeability to values. Here again we can see the importance of diversity as people look at hypotheses, results and methods from different perspectives.

Some fear that by admitting that scientists are not neutral actors in the pursuit of knowledge, they risk their credibility, giving lee‐way to science deniers, anti‐vaxxers, climate crisis deniers and pseudoscientists. This is a valid concern that even more justifies discussions among the natural scientists about the philosophical implications of their work.

The meaning of “facts” and “truth” has been shifting recently, and the risk of social regress is real. We have an increasing public health problem with infectious diseases in part caused by the anti‐vaccine movement that has scared parents, whereas in other parts of the world, mothers walk miles barefoot to make sure their kids are vaccinated and eradication of diseases is a pursuable victory. How can we respond to so‐called “post‐truth” or even “post‐science”? The Oxford Dictionary defines “post‐truth” as a “term relating to or denoting circumstances in which objective facts are less influential in shaping public opinion than appeals to emotion and personal belief”. But what is an objective fact, after all?

The answer to the post‐truth challenge of scientific evidence and its impact on the progress of society cannot rely on authoritative and dogmatic attitudes. This attitude does not build trust among the public but rather magnifies the suspicion that science has incorporated a religion‐like totalitarian attitude. Philosopher Bruno Latour argues that showing the public how scientific evidence is built—including the slowness and flaws of the process—is more convincing than just merely stating data and evidence 9. The only way forward is to be open—which includes being vulnerable.

This recent but urgent challenge requires a delicate balance from scientists and science communicators alike. Early career researchers should have more time for reflection about this. The awareness of the post‐truth challenge of scientific evidence should be an incentive for institutions to invest in strategic science outreach and for educators to reflect on how science is being taught. Could it be more important to teach from an early age about the multi‐dimensions of scientific evidence than about the theory of gravitation? How can we teach fact checking in an era of rising bogus scientific papers? It is important to note that showing the public how scientific evidence is built is not all about the flaws of the process: it is also about the beauty of inquiry in opposition to dogma. As Kathleen Higgins put it, society should be reminded of “the social mission of science” and of its “intellectual virtues: critical thinking, sustained inquiry and revision of beliefs on the basis of evidence” 10.

## Conclusion and perspective

Finally, the debate about the nature of the relationship between science and values and its advantages and disadvantages will not end soon, and hopefully never end at all. Its relevance is unquestionable as it is its complexity. This should not be a barrier to discussion, which ought to be open to all intervenient in the scientific process, but also to society as a whole.

The answer to the post‐truth challenge of scientific evidence and its impact on the progress of society cannot rely on authoritative and dogmatic attitudes.

I would like to advocate a non‐pessimistic perspective, a confidence in a science that can become more critical and less technic‐driven, and thereby taking advantage of the positive consequences which interference of values brings. Science is made by humans, humans who are driven by curiosity and the quest for knowledge, which include the critique of oneself. May these drivers be put into spotlight, to protect scientific endeavour from a competitive, mass production focused sometimes culturally unhealthy academia, and make sure that we not blindly serve the interests of progress. “And therefore, three cheers for physics! And still louder cheers for that which impels us to it ‐ our honesty” (Friedrich Nietzsche, Die fröhliche Wissenschaft 1882).
